# Aspirin promotes bone marrow mesenchymal stem cell-based calvarial bone regeneration in mini swine

**DOI:** 10.1186/s13287-015-0200-4

**Published:** 2015-10-31

**Authors:** Yu Cao, Jimin Xiong, Shenghui Mei, Fu Wang, Zhigang Zhao, Songlin Wang, Yi Liu

**Affiliations:** Department of General Dentistry, School of Stomatology, Capital Medical University, Beijing, P. R. China; Laboratory of Tissue Regeneration and Immunology and Department of Periodontics, Beijing Key Laboratory of Tooth Regeneration and Function Reconstruction, School of Stomatology, Capital Medical University, Tian Tan Xi Li No.4, Beijing, 100050 P. R. China; Department of Pharmacy, Beijing Tiantan Hospital, Capital Medical University, Beijing, P. R. China; Molecular Laboratory for Gene Therapy and Tooth Regeneration, Beijing Key Laboratory of Tooth Regeneration and Function Reconstruction, School of Stomatology, Capital Medical University, Beijing, P. R. China

**Keywords:** Mesenchymal stem cells, Aspirin, Bone regeneration, Mini swine

## Abstract

**Introduction:**

Stem cells have great therapeutic potential due to their capacity for self-renewal and their potential for differentiating into multiple cell lineages. It has been recently shown that the host immune system has fundamental effects on the fate of transplanted mesenchymal stem cells during bone repair, where the topical administration of aspirin is capable of improving calvarial bone repair in rodents by inhibiting tumor necrosis factor-α (TNF-α) and interferon-γ (IFN-γ) production. This study investigates whether aspirin is capable of accelerating the regenerative potential of bone marrow mesenchymal stem cells (BMSC) in a mini swine calvarial bone defect model.

**Methods:**

Calvarial bone defects (3 cm × 1.8 cm oval defect) in mini swine were treated with BMSC pretreated with 75 μg/ml aspirin for 24 h seeded onto hydroxyaptite/tricalcium phosphatel (HA/TCP), or with BMSC with HA/TCP, or with HA/TCP only, or remained untreated. Animals were scanned with micro-computed tomography (microCT) at 2 days and 6 months postsurgery and were sacrificed at 6 months postsurgery with decalcified tissues being processed for histomorphometric examination. The cytokine levels, including TNF-α and IFN-γ, were measured by enzyme-linked immunosorbent assay (ELISA).

**Results:**

Aspirin at 75 μg/ml promoted the osteogenesis of BMSC *in vitro* and *in vivo*, shown by Alizarin Red staining and new bone volume in the nude mice transplantation model (*p* < 0.01), respectively. Defects treated with aspirin-BMSC showed significantly greater new bone fill compared with other three groups at 6 months postsurgery (*p* < 0.01). Aspirin-BMSC treatment has significantly decreased the concentration of TNF-α and IFN-γ (*p* < 0.05).

**Conclusions:**

The present study shows that BMSC pretreated with aspirin have a greater capacity to repair calvarial bone defects in a mini swine model. The results suggest that the administration of aspirin is capable of improving BMSC-mediated calvarial bone regeneration in a big animal model.

**Electronic supplementary material:**

The online version of this article (doi:10.1186/s13287-015-0200-4) contains supplementary material, which is available to authorized users.

## Introduction

Adult or mesenchymal stem cells (MSC) are plastic adherent stromal cells found in special tissues and organs of human adults. With the capacity for self-renewal and multi-lineage differentiation, they are considered a promising cell source for tissue engineering, as they are easily accessible and not associated with ethical issues in relation to their use [1–5]. It has been widely accepted that MSC-based therapy has shown significant improvement of tissue regeneration in pre-clinical models and clinical trials. Despite the recent progress in MSC-based tissue regeneration in the last few decades, a major challenge remains how to restore new bone formation following disease or insult with the high quality and the bone volume that meet the needs of the body [6–9].

Recently, considerable interest has developed that the host immune imbalance accounts for, at least in part, the imbalance in bone remodeling that occurs in various bone disorders, such as arthritis and periodontitis, although microbial infection is thought to be one of the dominant factors in the initiation of periodontitis. As the immune imbalance might be one of the major factors in initiating the previously mentioned diseases, studies have been conducted aiming to manipulate the immune system of susceptible individuals. Studies have been carried out to investigate the interactions of MSC and the host immune system. Compared with embryonic stem cells, MSC have been shown to be low immunogenic, thus allogeneic MSC are able to escape the immune surveillance of the body and contribute to tissue regeneration [10–12]. On the other hand, we have recently shown that the host immune system has fundamental effects on the fate of transplanted MSC during bone remodeling, where TNF-α and IFN-γ produced by proinflammatory T cells play a critical role [13–15]. Intriguingly, the topical administration of aspirin, alternatively the systemic infusion of regulatory T cells, is capable of inhibiting TNF-α and IFN-γ production and, therefore, improve calvarial bone repair in rodents [13]. Compared with the systemic infusion of regulatory T cells, the topical administration of aspirin has more advantages from the safety aspects, since aspirin has been used as a non-steroidal anti-inflammatory agent (NSAID) for decades with a known side-effect profile. Moreover, the use of aspirin at the sites of tissue damage is less technically challenging than the systemic infusion of regulatory T cells and, therefore, can be easily accepted by clinical practitioners and patients.

Aspirin has recently been shown to regulate the balance between bone resorption and bone formation in ovariectomized-induced osteoporosis [16] and to accelerate bone repair in rodents [10]. It is imperative to investigate the safety and efficacy of aspirin-pretreated BMSC in a big animal model before clinical trials are initiated. In the present study, we examined whether aspirin-pretreated BMSC is capable of accelerating the healing process of calvarial bone defects in a mini swine model.

## Methods

### Animals

A total of 14 miniature pigs were used in the present study. Twelve inbred male miniature pigs (for calvarial defect surgery, 12 months of age) and two inbred female miniature pigs (for cell culture, three to four months of age) were supplied by the Institute of Animal Science of the Chinese Agriculture University (Beijing, China) and housed under conventional conditions. The study was conducted following the approved guidelines set by the Animal Ethics Committee of the School of Stomatology, Capital Medical University (Beijing, China). All animal experiments were performed under the institutionally approved protocols for the use of animal research (Capital Medical University # 2012-x-53).

### Isolation and culture of mini swine BMSC

Bone marrow aspirates were obtained from the posterior iliac crest of two inbred female mini swine under the approved guidelines set by the Animal Ethics Committee of the School of Stomatology, Capital Medical University (Beijing, China). Bone marrow mononuclear cells were prepared as described previously [17]. Primary bone marrow-derived mesenchymal stem cell (BMSC) cultures were established in tissue culture flasks and maintained in the cell growth medium. The cell growth medium contained alpha minimum essential medium (α-MEM, Invitrogen, Carlsbad, CA, USA.) supplemented with 10 % fetal calf serum, 100 μM L-ascorbic acid 2-phosphate, 2 mM L-glutamine (Biosource, Invitrogen), 100 U/ml penicillin and 100 μg/ml streptomycin in a humidified atmosphere (37 °C, 5 % CO_2_).

### Flow cytometric analysis

Flow cytometric analysis was performed as previously described [18]. Briefly, swine BMSC at passage three, treated with or without 75 μg/ml aspirin for 24 h, were detached and incubated with primary antibodies, including CD146 (MCAM, melanoma cell adhesion molecule) (BD Biosciences, San Jose, CA, USA), CD90 (THY-1, Thy-1 cell surface antigen) (BD Biosciences), CD31 (PECAM1, platelet/endothelial cell adhesion molecule 1) (BD Biosciences), HLA-DR (human leukocyte differentiation antigen class II) (Biolegend, San Diego, CA, USA) or corresponding isotype-matched control antibodies and fluorescein isothiocyanate (FITC)-conjugated secondary antibodies. Samples were washed, fixed with fluorescence-activated cell sorting (FACS) Fix and analyzed using a flow cytometer (Calibur, BD, Franklin Lakes, NJ, USA).

### Cell differentiation assay *in vitro*

Osteogenic and adipogenic differentiation assays were performed as previously reported [18, 19]. The calcification of extracellular matrix and lipid-laden droplets were detected using Alizarin Red and Oil Red O staining, respectively. The gene expressions of Runx2 and osteopontin were assayed by real time RT-PCR. The cells cultured in the growth medium (α-MEM supplemented with 10 % fetal calf serum, 100 μM L-ascorbic acid 2-phosphate, 2 mM L-glutamine and 100 U/ml penicillin/100 μg/ml streptomycin) were used as the control in the differentiation assay. GAPDH was used as a housekeeping control gene against which samples were normalized. All mRNA quantification data represent the mean ± standard error of the mean (SEM) of triplicate experiments normalized to the house-keeping gene GAPDH. All mRNA quantification data are presented as the fold changes in the expression of the gene of interest in the osteoinductive conditions to that in the control conditions.

### Cell proliferation assay *in vitro*

The effect of aspirin on swine BMSC proliferation was assessed using the MTT (3-[4,5-dimethylthiazol-2-yl]-2, 5-diphenyl tetrazolium bromide) assay. *Ex vivo* expanded swine BMSC were seeded at passage three (1.0 × 10^4^ cells/well) in triplicate using a 96-well flat-bottom plate (Costar, Cambridge, MA, USA) and maintained in 100 μl medium with aspirin (50, 75, 100, 150 or 200 μg/ml) or standard culture medium for five days. Cells were treated with 5 mg/ml of MTT reagent (Sigma-Aldrich, St. Louis, MO,USA) and incubated at 37 °C for 4 h. After cells were washed twice in PBS and treated with dimethyl sulfoxide, the absorbance in each well was measured at a wavelength of 490 nm using an automatic enzyme-linked immunosorbent assay (ELISA) reader (ELx800; BioTek Instruments Inc., Winooski, VT, USA).

### Cell growth curve assays

BMSCs were seeded in 60-mm plates at a density of 1.0 × 10^4^ cells/plate for cell growth curve assay. Cells were counted at 2, 3, 4, 5, and 6 days after seeding. Cells were digested with 0.25 % trypsin (Invitrogen), resuspended in 1 ml PBS and counted with an automated cell counter (TC10TM, Bio-Rad Laboratories, Hercules, California USA). An equivalent volume of trypan blue was added to the cell suspension to exclude nonviable cells.

### Alizarin red staining

BMSCs were grown in osteogenic-inducing medium, which contained the cell growth medium supplemented with 2 mM β-glycerophosphate, 1.8 mM KH_2_PO_4_, and 10 nM dexamethasone. To detect mineralization, cells were induced for three weeks, fixed with 70 % ethanol, and stained with 2 % Alizarin Red (Sigma-Aldrich). To determine calcium content quantitatively, cells stained with Alizarin Red were destained with 10 % cetylpyridinium chloride in 10 mM sodium phosphate for 30 min at room temperature. The calcium concentration was determined by measuring absorbance at 562 nm on a multiplate reader and comparing the reading to a standard calcium curve, constructed with calcium diluted in the same solution. The final calcium level in each group was normalized to the total protein on centration detected in a duplicate plate [20].

### Transplantation of BMSC into immunocompromised mice

Approximately 4.0 × 10^6^ BMSC, treated with or without 75 μg/ml aspirin for two days, were mixed with hydroxyapatite/tricalcium phosphate (HA/TCP) ceramic particles (40 mg; Engineering Research Center for Biomaterials, Sichuan University, China) as a carrier and subcutaneously implanted into the dorsal surface of eight- to ten-week-old immunocompromised mice. Xenogenic transplants were harvested at week 8 and stained with hematoxylin and eosin (H & E) staining before histological sections were analyzed for statistical evaluation.

### Generation of mini swine calvarial bone defect and transplantation of BMSC to the calvarial bone defect

The present study was performed under the approved guidelines of the Ethics Committee of the School of Stomatology, Capital Medical University, Beijing. The calvarial bone defect was created as previously described [21, 22]. Twelve inbred male miniature pigs (12 months of age) were supplied for calvarial defect surgery. Two oval defects (3 cm × 1.8 cm) were created in each animal; a total of 24 calvarial defects were generated in 12 miniature pigs. The defects were randomly assigned to four different groups and treated as following (six defects per group): (1) BMSC (1.0 × 10^6^) treated with 75 μg/ml aspirin for 24 h using HA/TCP as carrier, were transplanted into calvarial defects; (2) BMSC (1.0 × 10^6^) using HA/TCP as carrier, were transplanted into calvarial defects; (3) calvarial defects were filled with 40 mg HA/TCP only; and (4) calvarial defects were filled with nothing. The bone defects were then covered with absorbable gelatin sponges (Jinling Pharmaceutical CO., LTD, Nanjing, China). The defects that were filled with HA/TCP + BMSC treated with 75 μg/ml aspirin were covered with absorbable gelatin sponges with 75 μg/ml aspirin, while other groups were covered with absorbable gelatin sponges only. According to the manufacturer, the gelatin sponge is fully absorbed within four to six weeks.

### Evaluation of the release of aspirin in the gelatin sponge

To evaluate the kinetics of aspirin release, we analyzed the concentration of aspirin and its product of metabolism, salicylic acid, in absorbable gelatin sponge at different time points. Aspirin (100113–201405, 99.8 % purity), salicylic acid (100106–201104, 99.9 % purity) and tinidazole [100336–200703, 99.9 % purity, internal standard (IS)] were purchased from National Institutes for Food and Drug Control (Beijing, China), HPLC grade methanol, acetonitrile, and trifluoroacetic acid were purchased from Thermo Fisher Scientific (Waltham, MA, USA). Ultrapure water was obtained from a Milli-Q water purification device (Millipore, Bedford, MA, USA). Chromatographic analysis was performed on a Dionex Ultimate U3000 chromatographic system (Waltham, MA, USA). Data were acquired and processed using Chromeleon software (version 7.0). Briefly, the implanted absorbable gelatin sponges with aspirin were removed from surgery sites and collected into 1.5 ml eppendorf tubes at different time points. The absorbable gelatin sponges were weighed before being cut into small pieces and then 200 μl methanol was added to extract the drugs and precipitate the protein. The mixture was vortex mixed for one min, ultrasonic dissolved for one min, centrifuged at 10,000 × *g* for two min, then 100 μl of the supernatant was mixed with 10 μl IS, vortex mixed for one min, centrifuged at 10,000 × *g* for two min, and then 20 μl of the supernatant was injected into the HPLC system. Chromatographic separation was achieved using Acclaim™ C18 column (150 × 4.6 mm, 5 μm particles, Thermo Scientific). Isocratic elution consisted of acetonitrile and water (28:72) (both contain 0.05 % trifluoroacetic acid). The flow rate was 1.0 ml/min, the injection volume was 20 μl and the oven temperature was set at 30 °C. Aspirin, salicylic acid and tinidazole were measured at 277 nm using a diode array detector.

### Quantitative and histological evaluation of regenerated bone

The calvarial bone samples were harvested at six months postsurgery. Bone specimens were fixed in 10 % buffered formalin. The specimens were decalcified and embedded in paraffin. Sections of 5- to 6-μm thickness from the embedded specimen were stained with H & E, Masson’s trichrome or methylene blue in the Institute of Dental Research, General Hospital of Chinese PLA (Beijing, China). The volume of newly-formed bone within each section was analyzed semi-quantitatively by histomorphometric techniques.

Masson’s trichrome staining was performed according to the manufacturer’s protocol (Cat No.26386, Electron Microscopy Science, Hatfield, PA, USA). Briefly, tissue sections were deparaffinized, stained with Bouin’s Fluid solution, cooled and washed in running tap water until the yellow color disappeared. The sections were then placed in Weigert’s hematoxylin, stained with Biebrich scarlet-acid fuchsin solution and washed in distilled water. The sections were placed in phosphomolybdic-phosphotungstic acid solution until the collagen was decolorized, before they were transferred directly (without rinse) to the aniline blue solution and washed with 1 % acetic acid. With this method, blue staining indicates regenerated bone, osteoid or collagen fibers, while red staining indicates mature bone and orange staining indicates erythrocyte.

### Cytokine levels in mini swine calvarial bone tissues

The cytokine levels in the implants were measured by ELISA [13]. The implants of BMSC mixed with HA-TCP ceramic particles were harvested at day 30 after transplantation into calvarial bone defects. The cytokine concentrations, including TNF-α and IFN-γ (Invitrogen, Carlsbad, Canada), in the calvarial bone tissues were measured using a commercial ELISA kit according to the instructions of the manufacturer.

### Bone analysis by micro-computed tomography

Analysis was conducted as previously described. 3D microarchitecture of the calvarial bone samples was evaluated using micro-computed tomography (microCT) (110 kv, 19 mA) two days and six months postsurgery. The newly-formed bone density and bone thickness were measured and compared between different groups.

### Statistical analysis

Data analysis was carried out using SPSS10 statistical software. Data points are reported as the mean ± standard deviation (SD) or mean ± standard error of the mean (SEM). Statistical significance of (*) *p* ≤ 0.05 was determined using the unpaired Student t-testor one-way analysis of variance (ANOVA). Statistical analysis was performed as described using at least three biological replicates unless otherwise stated.

## Results

### Isolation and characterization of BMSC

To confirm the differentiation potential of swine BMSC, *ex vivo* expanded BMSC were subjected to osteogenic and adipogenic inductive culture conditions for four weeks. Alizarin Red-positive mineral nodules and Oil Red O-positive fat-laden droplets were formed in osteogenic and adipogenic culture conditions, respectively (data not shown).

### Aspirin has no effect on the immunophenotype of swine BMSC

To examine whether aspirin treatment affects the cell surface marker profile of BMSC, cells were treated with 75 μg/ml aspirin for 24 h before their immunophenotype was investigated using flow cytometry. Aspirin-treated swine BMSC had a compatible cell surface expression pattern with untreated cells (data not shown), which showed negativity for an endothelial cell surface marker, CD31 (platelet endothelial cell adhesion molecule-1/PECAM-1) and high positivity for CD90 (cell surface markers associated with stem cells). Aspirin treatment did not affect the cell surface expression of STRO-1 (early mesenchymal stem cell marker) (data not shown).

### The effect of aspirin on BMSC proliferation

**Fig. 1:**
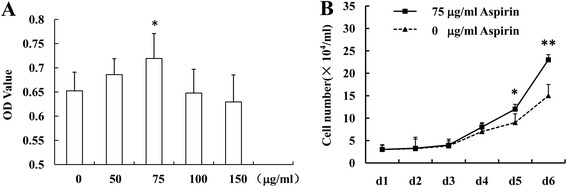
Aspirin at 75 μg/ml enhances BMSC proliferation. Aspirin at 75 μg/ml enhanced BMSC proliferation, while aspirin at high concentrations displayed an inhibitory effect on cell proliferation, shown by MTT assay a. The cell growth curve assay showed that aspirin at 75 μg/ml enhanced BMSC proliferation **b**. The results are representative of at least three independent experiments. Results are expressed as mean ± standard deviation (SD) and statistical significance is shown as (*) *p* < 0.05 or (**) *p* < 0.01. *BMSC* bone marrow mesenchymal stem cells

BMSC were treated with aspirin at various concentrations (50, 75, 100, and 150 μg/ml) before the rate of cell proliferation was examined with the MTT assay. Aspirin at 75 μg/ml was shown to enhance BMSC proliferation (Fig. 1a). Aspirin at high concentrations (100 and 150 μg/ml) seemed to display an inhibitory effect on cell proliferation but these results were not statistically significant (Fig. 1a). The cell growth curve assay also confirmed that aspirin at 75 μg/ml enhanced BMSC proliferation (Fig. 1b).

### Aspirin stimulates the osteogenesis of BMSC *in vitro* and *in vivo*

**Fig. 2:**
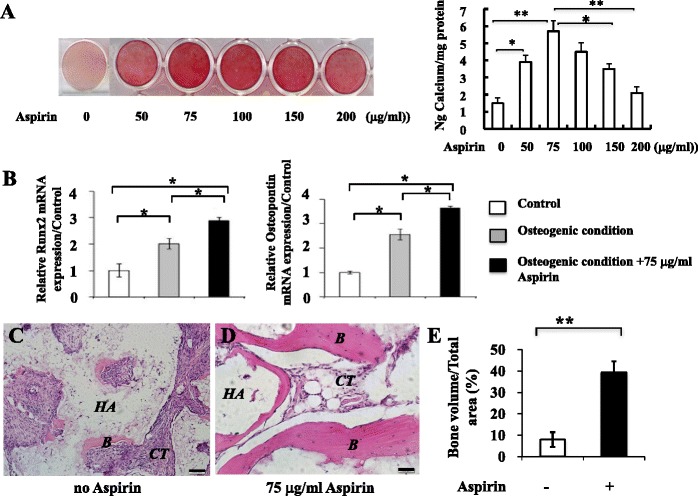
Aspirin stimulates the osteogenesis of BMSC *in vitro* and *in vivo*. BMSC treated with different concentrations of aspirin increased the capability of forming Alizarin red-positive calcified deposits **a**. BMSC treated with 75 μg/ml aspirin showed the up-regulation of bone related genes (Runx2 and osteopontin) **b**. Aspirin treatment significantly stimulated BMSC-mediated bone formation in immunocompromised mice transplants **c**-**e**. The results are representative of at least three independent experiments. Results are expressed as mean ± standard deviation (SD) and statistical significance is shown as (*) *p* < 0.05 or (**) *p* < 0.01. Scale bars = 100 μm. *B* bone, *HA* hydroxyapatite/tricalcium phosphate, *CT* connective tissues, *BMSC* bone marrow mesenchymal stem cells

We next explored whether aspirin treatment would promote the osteogenic potential of swine BMSC. As shown in Fig. 2a, when BMSC were subjected to osteogenic inductive conditions, aspirin treatment at concentrations of 50, 75, 100, 150, and 200 μg/ml increased the capability of forming Alizarin red-positive calcified deposits. This was confirmed by the up-regulation of bone-related genes (Runx2 and osteopontin) after BMSC were treated with 75 μg/ml aspirin in osteoinductive conditions for two weeks (Fig. 2b) (*P* < 0.05).

To investigate the effect of aspirin treatment on the mineral forming capacities of swine BMSC *in vivo*, using a “gold standard” for assessing stem cell characteristics, BMSC treated with 75 μg/ml aspirin or untreated cells were transplanted into immunocompromised mice using HA/TCP as a carrier and samples were recovered at week 8. Aspirin treatment significantly stimulated BMSC-mediated bone formation in nude mice as shown in Fig. 2c, d, and e (*p* < 0.05). These data collectively indicate that aspirin at the concentration of 75 μg/ml promotes BMSC-based mineral formation both *in vitro* and *in vivo*.

### Aspirin promotes BMSC-based calvarial bone regeneration in mini swine

**Fig. 3:**
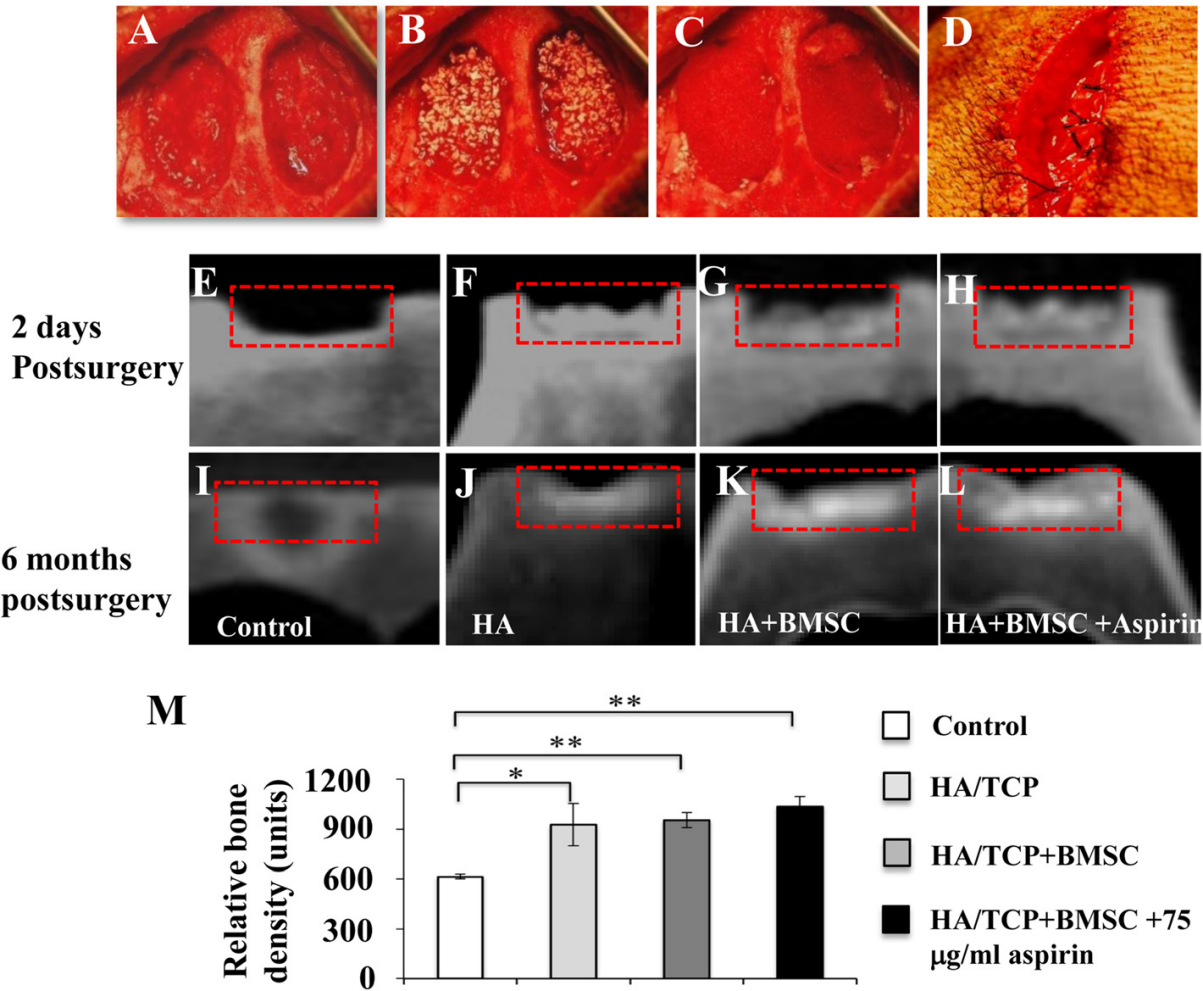
Aspirin promotes BMSC-based calvarial bone regeneration in mini swine shown by microcomputed tomography (microCT) images. Two freshly created calvarial bone defects were created in each mini swine before they were filled with nothing, HA/TCP, HA/TCP + BMSC treated with or without 75 μg/ml aspirin **a**-**d**. MicroCT images of calvarial defect areas were captured two days **e**-**h** and six months postsurgery **i**-**l**, showing new bone generation in the untreated group **e** and **i**, HA/TCP group **f** and **j**, HA/TCP + BMSC **g** and **k** and HA/TCP + BMSC-aspirin **h** and **l**. Quantitative examination of microCT images illustrated relative new bone density between different groups six months postsurgery **m**. Results are expressed as mean ± standard deviation (SD) and statistical significance is shown as (*) *p* < 0.05 or (**) *p* < 0.01. *BMSC* bone marrow mesenchymal stem cells, *HA/TCP* hydroxyapatite/tricalcium phosphate

As aspirin is capable of promoting the osteogenesis of BMSC, we hypothesized that aspirin treatment, together with BMSC, might improve the healing process of calvarial bone defects in mini swine. Calvarial bone defects were freshly created in mini swine (Fig. 3a-d) before they were filled with nothing, HA/TCP, or HA/TCP + BMSC treated with or without 75 μg/ml aspirin for 24 h. To investigate whether aspirin treatment has any potential side effects on biochemistry values in mini swine, whole blood samples were collected for biochemical testing. Data illustrated that aspirin treatment did not significantly alter the biochemistry profile of mini swine (data not shown), suggesting the safety of using aspirin-treated BMSC for promoting bone formation *in vivo*. Experiments on the kinetics of aspirin release in the absorbable gelatin sponge have shown that aspirin or salicylic acid could almost not be detected after 3 h (Additional file 1: Figure S1).

Two days postsurgery, the operative defects could be easily identified in microCT images showing the margin of the defects (Fig. 3e-h). At six months following surgery, the margin of the defects was not clearly identifiable as the formation of mineralized tissue (Fig. 3i-l). Quantitative examination of microCT images illustrated that at the six-month time point, the untreated group showed significantly lower new bone density than the other three groups (*p* < 0.05), while the HA/TCP + BMSC-aspirin group did not show higher new bone density than the HA/TCP+BMSC or HA/TCP group (Fig. 3m).

**Fig. 4:**
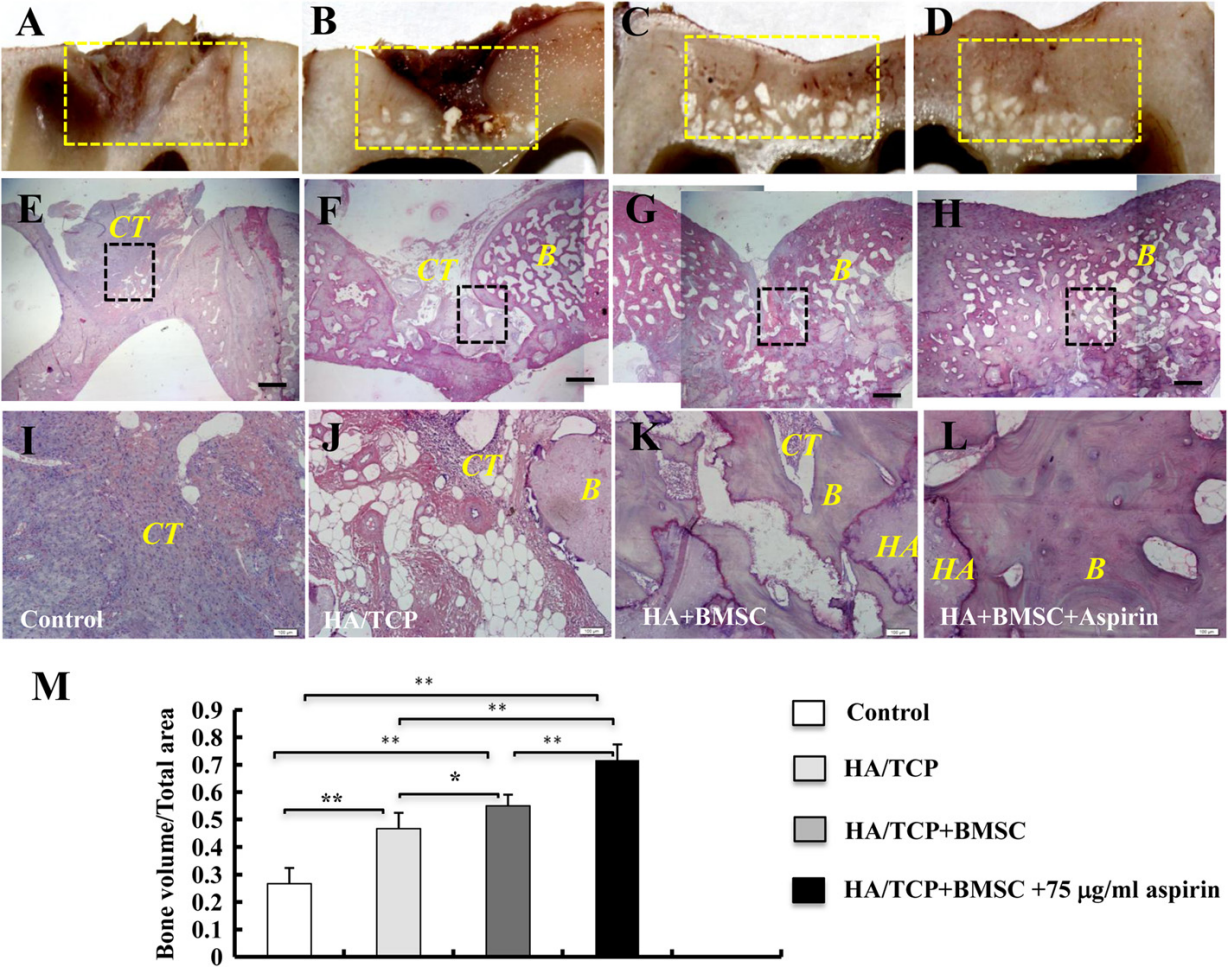
Aspirin promotes BMSC-based calvarial bone regeneration in mini swine. Calvarial bone specimens, treated with nothing **a**, HA/TCP **b**, HA/TCP + BMSC **c** or HA/TCP + BMSC-aspirin **d**, were retrieved six months postsurgery. Sections were stained with H&E **e**-**l**. Images were captured at the lower **e**-**h** and higher magnification **i**-**l**. Semi-quantitative analysis of the percentage of new bone formation between different groups was based on the results of H&E staining **m**. The results are representative of at least three independent experiments. Results are expressed as mean ± standard deviation (SD) and statistical significance is shown as (*) *p* < 0.05 or (**) *p* < 0.01. Scale bars = 1 cm in **e**-**h**; scale bars = 100 μm in **i**-**l**. *B* bone, *HA* hydroxyapatite/tricalcium phosphate, CT connective tissues, *BMSC* bone marrow mesenchymal stem cells, *H & E* hematoxylin and eosin

When calvarial bone specimens were retrieved 12 months postsurgery, minimal formation of mineralized tissues was observed in the untreated group (Fig. 4a), while limited new bone generation was noted in the HA/TCP group (Fig. 4b). A greater degree of bone-like tissue formation was noted in defects treated with HA/TCP+BMSC-aspirin (Fig. 4d), compared with those treated with HA/TCP+BMSC (Fig. 4c).

**Fig. 5:**
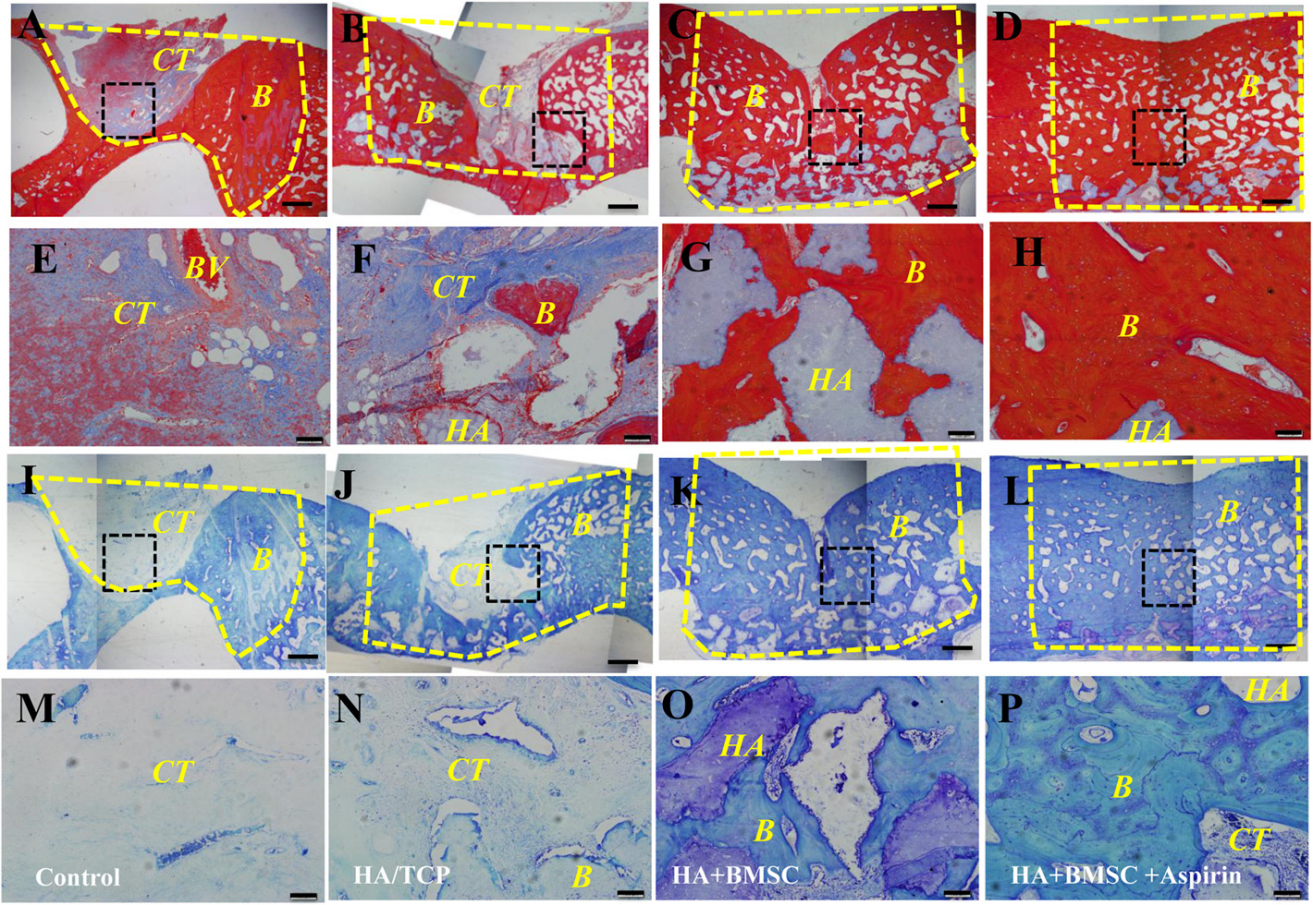
Aspirin promotes BMSC-based calvarial bone regeneration in mini swine shown by histomorphometric analysis. Calvarial bone specimens, treated with nothing, HA/TCP, HA/TCP + BMSC or HA/TCP + BMSC-aspirin, were retrieved six months postsurgery. Sections were stained with Masson’s trichrome **a**-**h** or methylene blue **i**-**p**. Images were captured at the lower **a**-**d** and **i**-**l** and higher magnification **e**-**h** and **m**-**p**. Scale bars = 1 cm in **a**-**d** and **i**-**l**; scale bars = 100 μm in **e**-**h** and **m**-**p**. *B* bone; *HA* hydroxyapatite/tricalcium phosphate, *CT* connective tissues, *BMSC* bone marrow mesenchymal stem cells

Calvarial bone specimens were retrieved and sections were sent for histological examination using H&E (Fig. 4e-l), Masson’s trichrome (Fig. 5a-h) or methylene blue staining (Fig. 5i-p). Images were captured at the lower magnification showing the whole defect area (Figs. 4e-h, 5a-d and 5i-l). Untreated defects appear to be filled with fibrous tissue with minimal bone formation (Figs. 4e, 5a, and 5i), while HA/TCP-treated defects exhibit formation of moderate amount of mineralized tissues (Figs. 4f, 5b, and 5j). The HA/TCP+BMSC-aspirin group displayed almost full restoration of the defect (Figs. 4h, 5d and 5l), with improvement of formation of newly-formed bone compared to the control HA/TCP+BMSC group (Figs. 4g, 5c and 5k). At higher magnification (Figs. 4i-l, 5e-m, and 5m-p), untreated defects were filled with fibrous tissue (Figs. 4i, 5e and 5m), while limited new bone formation was noted in the HA/TCP group (Figs. 4j, 5f and 5n). A moderate amount of newly-formed bone was noted in defects treated with HA/TCP+BMSC (Figs. 4k, 5g, and 5o). The HA/TCP+BMSC-aspirin group demonstrated formation of an abundant amount of mineralized tissue (Figs. 4l, 5h, and 5p). The presence of HA/TCP particles was also noted. Semi-quantitative analysis of the percentage of new bone formation showed that the HA/TCP + BMSC-aspirin group demonstrated a statistically higher percentage of mineralized matrix formation at the regenerated defect site (71.6 ± 5.77 %) compared with the HA/TCP + BMSC group (55.0 ± 4.08 %), the HA/TCP group (43.3 ± 5.75 %), or the control group (26.6 ± 5.75 %) (*n* = 6 in each group) (*P* < 0.01) (Fig. 4m).

### Aspirin treatment reduced the concentration of proinflammatory cytokines in calvarial bone defects

**Fig. 6:**
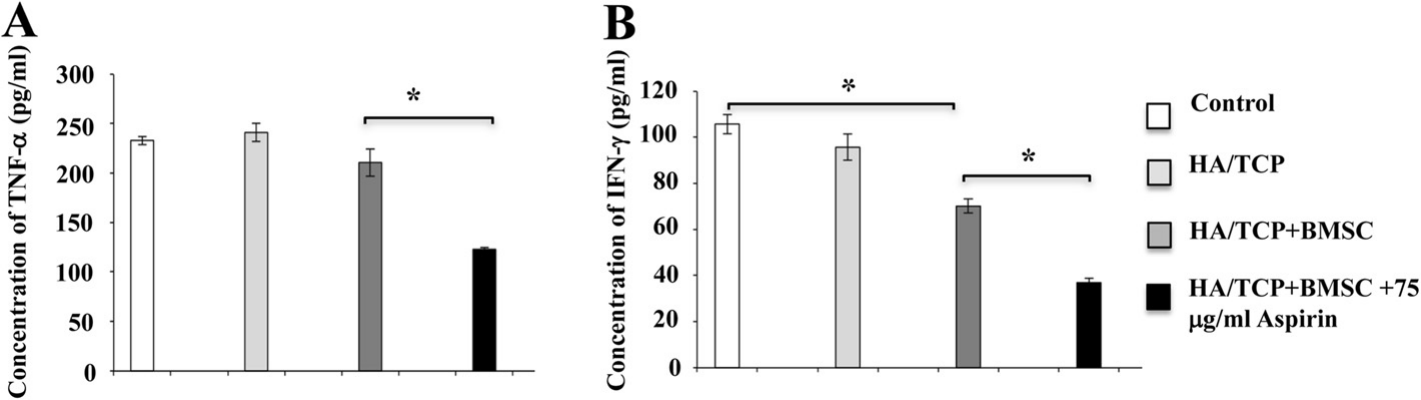
Aspirin treatment reduced levels of tumor necrosis factor-α (TNF-α) and interferon-γ (IFN-γ) in mini swine calvarial bone defects. Aspirin pretreatment significantly decreased the concentration of TNF-α **a** and IFN-γ **b** compared to the HA/TCP-BMSC group. BMSC treatment reduced the concentration IFN-γ in the calvarial bone defect **b** but not that of TNF-α **a**. The results are representative of at least three independent experiments. Results are expressed as mean ± standard deviation (SD) and statistical significance is shown as (*) *p* < 0.05 or (**) *p* < 0.01. *HA/TCP* hydroxyapatite/tricalcium phosphate, *BMSC* bone marrow mesenchymal stem cells

As we have recently reported that BMSC-mediated bone formation is negatively correlated with the concentrations of TNF-α and IFN-γ [13], we investigated the concentration of TNF-α and IFN-γ in mini swine calvarial bone defects. Aspirin pretreatment significantly decreased the concentration of TNF-α (Fig. 6a) and IFN-γ (Fig. 6b) compared to the HA/TCP-BMSC group (*p* < 0.05), while BMSC treatment significantly reduced the concentration of IFN-γ in the calvarial bone defect (Fig. 6b) (*P* < 0.05) but not that of TNF-α (Fig. 6a).

## Discussion

Although MSC-based therapy has shown promising outcomes of tissue regeneration in pre-clinical models and clinical trials in the last few decades, a major challenge remains how to restore new bone formation following disease or insult with the high quality and bone volume that meet the needs of the body. The present study is, to our knowledge, the first report that the administration of aspirin, coupled with bone marrow-derived mesenchymal stem cells, is capable of promoting bone repair in a big animal model. In addition, aspirin has been used as a non-steroidal anti-inflammatory drug (NSAID) for decades with a known side-effect profile. Therefore, the topical administration of aspirin holds greater advantages from the safety perspective, compared with other strategies to promote the osteogenesis of stem cells, such as the systemic infusion of regulatory T cells, the use of genetically modified cells and the treatment of stem cells with growth factors. Collectively, the present study sheds light on the therapeutic effects of aspirin-treated MSC in a big animal model and encourages clinical trials for bone-related disorders, such as bone fractures, periodontitis, arthritis, and orofacial deformity.

Aspirin (acetylsalicylic acid or salicylic acid) is a NSAID drug that has been widely used for a variety of clinical applications, such as to relieve pain, to reduce fever and inflammation, to treat and prevent common cardiovascular disorders, such as heart attacks and strokes, and as an anti-inflammatory medication. Due to its involvement in multiple biological pathways, aspirin may have as-yet-unknown benefit(s) in a variety of conditions that deserve further investigation [23–26].

Studies have been focused on the roles of aspirin in the process of bone metabolism for the last decade. Epidemiologic studies revealed that current aspirin users had significantly higher bone mineral density compared with non-users according to quantitative computed tomography data [23]. A recent study has highlighted the positive effect of aspirin in the treatment of ovariectomy-induced osteoporosis through activating osteoblasts by increasing telomerase activity and inhibiting osteoclasts [16]. Due to its involvement in multiple biological pathways including inhibiting cyclo-oxygenase-1 (COX-1), COX-2 and prostaglandin 2 (PG_2_), it is very difficult to identify the exact mechanisms of its roles in bone remodeling [16]. The underlying mechanisms of the effect of aspirin on bone metabolism appear to be correlated with the increased expression of osteogenic genes including Runx2 (a master gene for osteogenic differentiation), alkaline phosphatase and osteocalcin [16]. Additionally, *ex vivo* aspirin treatment was capable of accelerating degradation of phospho-β-catenin, resulting in an increased level of WNT signaling, a recognized pathway in osteogenesis [27].

The present study lends support to the notion that aspirin treatment is capable of promoting osteogenesis both *in vitro* and in the mice transplantation model. This is consistent with our previous study showing that aspirin has a direct positive effect on the bone forming ability of BMSC [13]. It should be noted that both *in vitro* tissue culture and the mice transplantation model represent a microenvironment in the absence of inflammation or with low inflammation levels. On the other hand, an inflammatory microenvironment is present in various disease settings, either in an acute or chronic manner, such as periodontitis, bone fractures and orofacial deformity. This inflammatory microenvironment has a fundamental impact on the regenerative capacities of both endogenous [28] and exogenous MSC [13]. It is imperative to investigate the effect of aspirin pretreatment in a microenvironment with inflammatory cell infiltration where BMSC osteogenic capacity has been compromised, such as freshly created calvarial bone defects, preferably in a big animal model as in the present study.

In the present study, absorbable gelatin sponges with or without aspirin were used to cover the defect area. We analyzed the concentration of aspirin and salicylic acid in transplanted HA/TCP at different time points. However, the concentrations of aspirin and salicylic acid were too low to be assayed. This may be due to the fact that the gelatin sponges had been squeezed to make sure only a limited amount of aspirin was left in the sponges before the sponges were used to cover the defect area. This is to minimize the possibility that the treatment on one side of the animal might interfere with that on the other side, where the treatment on both sides of the animal might not be the same. In other words, this is to avoid aspirin on the experimental side interfering with the healing of the defects on the other side. Evaluation of the kinetics of aspirin and salicylic acid release showed that after 3 h, almost no aspirin or salicylic acid could be detected in gelatin sponges. This indicates that the gelatin sponges served as a barrier to stabilize the blood clot and transplantation in the defects area in the present study, rather than for aspirin to be slowly released.

A delicate host-parasite balance is thought to be interrupted in a variety of bone disorders, such as periodontitis and arthritis. For example, it has been accepted that host systemic conditions account for, at least in part, the imbalance in bone remodeling in the process of periodontitis, although the bacterial infection is thought to be one of the dominant factors. While the current therapeutic strategy for the management of periodontitis, scaling and root planing, is based on documented scientific literature, we believe that mechanical debridement in conjunction with anti-inflammatory agents might give favorable therapeutic effects in a variety of clinical settings. Previous studies have reported that aspirin is capable of inhibiting the production of TNF-α and IFN-γ [29]. Due to its immunomodulatory properties, aspirin has been used to treat patients with renal transplantation [30]. We have recently reported that aspirin is capable of reducing the concentrations of TNF-α and IFN-γ and rescuing the osteogenic deficiency of BMSC induced by proinflammatory cytokines [13]. In addition, studies have been conducted showing the efficiency of aspirin treatment in MSCs-mediated cell therapy for treating immune-related disorders, as aspirin is capable of promoting immunoregulatory properties of BMMSCs via the 15d-PGJ_2_/PPARγ/TGF-β1 pathway and aspirin-pretreated BMMSCs significantly ameliorated disease activity and colonic inflammation of dextran sodium sulfate (DSS)-induced experimental colitis in a mice model [31]. In this study, the concentrations of TNF-α and IFN-γ in the aspirin treatment group and the concentration of IFN-γ in the BMSC group were significantly decreased in the calvarial bone defects, which suggests the locally transplanted BMSC also have immunoregulatory properties in local sites.

## Conclusions

We have shown in the present study that aspirin-treated BMSC is capable of promoting calvarial bone regeneration in a big animal model. We postulated that the local administration of aspirin, coupled with MSC, has a twofold effect on tissue healing; one alleviating inflammatory response at sites of disease and the other promoting MSC-based regenerative capacities (both endogenous and exogenous MSC) through the suppression of TNF-α and IFN-γ. Data from a blood biochemistry test demonstrated no significant changes in animals treated with aspirin-BMSC. Coupled with the fact that aspirin has been used as a NSAID for decades with a known side-effect profile, the local administration of aspirin should possess fewer safety concerns compared with other strategies, such as the use of genetically modified stem cells or systemic infusion of regulatory T cells. Future clinical studies may seek to investigate the potential of aspirin-treated BMSC for treating bone-related disorders, such as bone fractures, periodontitis, arthritis, orofacial deformity and immune-related disorders.

## Box 1. About Yi Liu

Dr. Yi Liu is a Professor and Department Chair of the Department of Periodontics in Capital Medical University School of Stomatology, China. Dr. Liu earned a DDS degree from West China College of Stomatology, Sichuan University as well as a PhD from Capital Medical University School of Stomatology. She is a Standing Committee Member of the Chinese Stomatological Association of Periodontal Disease and a Committee Member of the Chinese Stomatological Association of Biomedical. Over the years, Dr. Liu is most noted for her research on mesenchymal stem cell-mediated oral and maxillofacial tissue regeneration and transformation medicine. More recently, she focuses on the mechanism of host immune system controlling tissue regeneration, and investigates the effective immune regulation methods to improve tissue regeneration. Dr. Liu has published more than 30 scientific articles in a variety of scientific journals.

## Note

This article is part of an ‘Emerging Investigators’ collection showcasing the work of early career investigators who have demonstrated growing leadership in the field of stem cells and regenerative medicine. Other articles in the series can be found online at http://stemcellres.com/series/emerginginvestigators

## Additional file

Additional file 1:
**Concentration of aspirin and salicylic acid in the gelatin sponge at different time points normalized to the weight of gelatin sponge.** Aspirin or salicylic acid could almost not be detected after 3 h. (JPEG 491 kb)

## Abbreviations

BMSC: Bone marrow mesenchymal stem cells
ELISA: Enzyme-linked immunosorbent assay
H&E: Hematoxylin and eosin
HA/TCP: Hydroxyapatite/tricalcium phosphate
IFN-γ: Interferon-γ
microCT: Micro-computed tomography
MSC: Mesenchymal stem cells
MTT: 3-[4,5-dimethylthiazol-2-yl]-2, 5-diphenyl tetrazolium bromide
NSAID: non-steroidal anti-inflammatory drug
PPARγ: Peroxisome proliferator-activated receptors gamma
TNF-α: Tumor necrosis factor-α

## References

[1] Bianco P, Riminucci M, Gronthos S, Robey PG (2001). Bone marrow stromal stem cells: nature, biology, and potential applications. Stem Cells..

[2] Friedenstein AJ, Chailakhyan RK, Latsinik NV, Panasyuk AF, Keiliss-Borok IV (1974). Stromal cells responsible for transferring the microenvironment of the hemopoietic tissues. Cloning in vitro and retransplantation in vivo. Transplantation.

[3] Owen M, Friedenstein AJ (1988). Stromal stem cells: marrow-derived osteogenic precursors. Ciba Found Symp..

[4] Pittenger MF, Mackay AM, Beck SC, Jaiswal RK, Douglas R, Mosca JD (1999). Multilineage potential of adult human mesenchymal stem cells. Science..

[5] Prockop DJ (1997). Marrow stromal cells as stem cells for nonhematopoietic tissues. Science..

[6] Caplan AI (2007). Adult mesenchymal stem cells for tissue engineering versus regenerative medicine. J Cell Physiol..

[7] García-Gómez I, Elvira G, Zapata AG, Lamana ML, Ramírez M, Castro JG (2010). Mesenchymal stem cells: biological properties and clinical applications. Expert Opin Biol Ther..

[8] Tasso R, Fais F, Reverberi D, Tortelli F, Cancedda R (2010). The recruitment of two consecutive and different waves of host stem/progenitor cells during the development of tissue-engineered bone in a murine model. Biomaterials..

[9] Bueno EM, Glowacki J (2009). Cell-free and cell-based approaches for bone regeneration. Nat Rev Rheumatol..

[10] Le Blanc K, Tammik C, Rosendahl K, Zetterberg E, Ringdén O (2003). HLA expression and immunologic properties of differentiated and undifferentiated mesenchymal stem cells. Exp Hematol..

[11] Zhang B, Liu R, Shi D, Liu X, Chen Y, Dou X (2009). Mesenchymal stem cells induce mature dendritic cells into a novel Jagged-2-dependent regulatory dendritic cell population. Blood..

[12] Majumdar MK, Keane-Moore M, Buyaner D, Hardy WB, Moorman MA, McIntosh KR (2003). Characterization and functionality of cell surface molecules on human mesenchymal stem cells. J Biomed Sci..

[13] Liu Y, Wang L, Kikuiri T, Akiyama K, Chen C, Xu X (2011). Mesenchymal stem cell-based tissue regeneration is governed by recipient T lymphocytes via IFN-γ and TNF-α. Nat Med..

[14] Su Y, Shi S, Liu Y (2014). Immunomodulation regulates mesenchymal stem cell-based bone regeneration. Oral Dis..

[15] Shi Y, Wei L, Wang Y, Ren G (2012). Stem cells deployed for bone repair hijacked by T cells. Cell Stem Cell..

[16] Yamaza T, Miura Y, Bi Y, Liu Y, Akiyama K, Sonoyama W (2008). Pharmacologic stem cell based intervention as a new approach to osteoporosis treatment in rodents. PLoS One..

[17] Gronthos S, Zannettino AC, Hay SJ, Shi S, Graves SE, Kortesidis A (2003). Molecular and cellular characterisation of highly purified stromal stem cells derived from human bone marrow. J Cell Sci..

[18] Xiong J, Mrozik KM, Gronthos S, Bartold PM (2012). Epithelial cell rests of Malassez contain unique stem cell populations capable of undergoing epithelial-mesenchymal transition. Stem Cells Dev..

[19] Jonsdottir-Buch SM, Lieder R, Sigurjonsson OE (2013). Platelet lysates produced from expired platelet concentrates support growth and osteogenic differentiation of mesenchymal stem cells. PLoS One..

[20] Fan Z, Yamaza T, Lee JS, Yu J, Wang SL, Fan G (2009). BCOR regulates mesenchymal stem cell function by epigenetic mechanisms. Nat Cell Biol..

[21] Fang D, Seo BM, Liu Y, Sonoyama W, Yamaza T, Zhang C (2007). Transplantation of mesenchymal stem cells is an optimal approach for plastic surgery. Stem Cells..

[22] Wang S, Liu Y, Fang D, Shi S (2007). The miniature pig: a useful large animal model for dental and orofacial research. Oral Dis..

[23] Carbone LD, Tylavsky FA, Cauley JA, Harris TB, Lang TF, Bauer DC (2003). Association between bone mineral density and the use of nonsteroidal anti-inflammatory drugs and aspirin: impact of cyclooxygenase selectivity. J Bone Miner Res..

[24] Shi S, Yamaza T, Akiyama K (2008). Is aspirin treatment an appropriate intervention to osteoporosis?. Fut Rheumatol..

[25] Lussana F, Squizzato A, Permunian ET, Cattaneo M (2014). A systematic review on the effect of aspirin in the prevention of post-operative arterial thrombosis in patients undergoing total hip and total knee arthroplasty. Thromb Res..

[26] Chen C, Akiyama K, Yamaza T, You YO, Xu X, Li B (2014). Telomerase governs immunomodulatory properties of mesenchymal stem cells by regulating FAS ligand expression. EMBO Mol Med..

[27] Shi S, Gronthos S, Chen S, Reddi A, Counter CM, Robey PG (2002). Bone formation by human postnatal bone marrow stromal stem cells is enhanced by telomerase expression. Nat Biotechnol..

[28] Liu D, Xu J, Liu O, Fan Z, Liu Y, Wang F (2012). Mesenchymal stem cells derived from inflamed periodontal ligaments exhibit impaired immunomodulation. J Clin Periodontol..

[29] Kwon MS, Shim EJ, Seo YJ, Choi SS, Lee JY, Lee HK (2005). Effect of aspirin and acetaminophen on proinflammatory cytokine-induced pain behavior in mice. Pharmacology..

[30] Grotz W, Siebig S, Olschewski M, Strey CW, Peter K (2004). Low-dose aspirin therapy is associated with improved allograft function and prolonged allograft survival after kidney transplantation. Transplantation..

[31] Tang J, Xiong J, Wu T, Tang Z, Ding G, Zhang C (2014). Aspirin treatment improved mesenchymal stem cell immunomodulatory properties *via* the 15d-PGJ2/PPARγ/TGF-β1 pathway. Stem Cells Dev..

