# Supplementary figures and images for: Aspirin promotes bone marrow mesenchymal stem cell-based calvarial bone regeneration in mini swine
Source: Stem Cell Res Ther. 2015 Oct 31;6:210. doi: 10.1186/s13287-015-0200-4 (PMC4628405; doi:10.1186/s13287-015-0200-4)

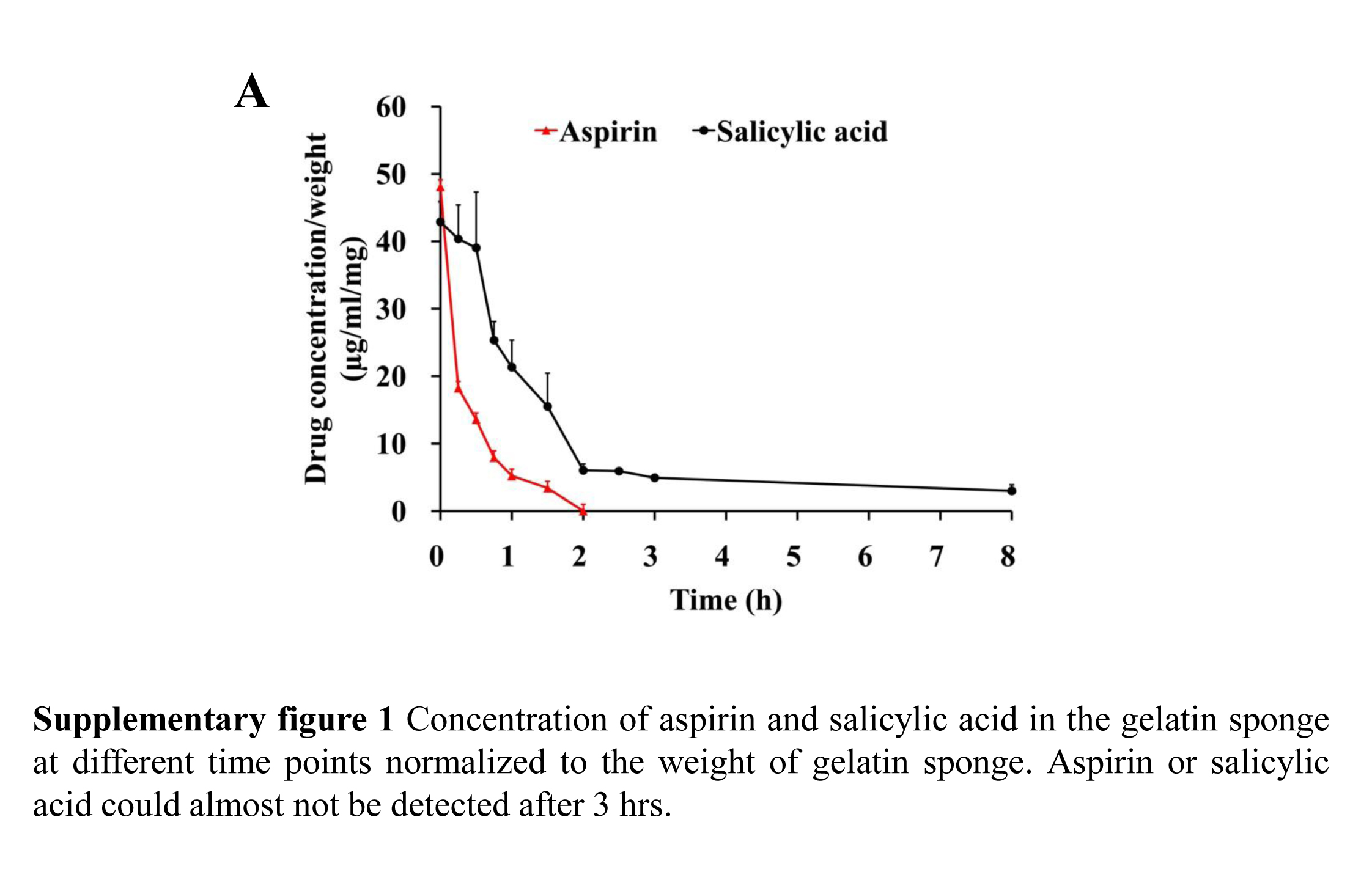

Supplement: Additional file 1: — Concentration of aspirin and salicylic acid in the gelatin sponge at different time points normalized to the weight of gelatin sponge. Aspirin or salicylic acid could almost not be detected after 3 h. (JPEG 491 kb) [file 13287_2015_200_MOESM1_ESM.jpeg]
